# Acute Pharmacological Effects and Oral Fluid Concentrations of the Synthetic Cannabinoids JWH-122 and JWH-210 in Humans After Self-Administration: An Observational Study

**DOI:** 10.3389/fphar.2021.705643

**Published:** 2021-08-19

**Authors:** Lucia Martínez, Nunzia La Maida, Esther Papaseit, Clara Pérez-Mañá, Lourdes Poyatos, Manuela Pellegrini, Simona Pichini, Mireia Ventura, Liliana Galindo, Francesco Paolo Busardò, Magí Farré

**Affiliations:** ^1^ Clinical Pharmacology Unit, Hospital Universitari Germans Trias i Pujol, Institut de Recerca Germans Trias i Pujol (HUGTiP-IGTP), Barcelona, Spain; ^2^ Clinical Pharmacology Department, Hospital Universitario La Paz, Madrid, Spain; ^3^ Department of Excellence of Biomedical Science and Public Health, University “Politecnica delle Marche”, Ancona, Italy; ^4^ Department of Pharmacology, Therapeutics and Toxicology, Universitat Autònoma de Barcelona, Cerdanyola del Vallés, Spain; ^5^ National Centre on Addiction and Doping, Istituto Superiore di Sanità, Rome, Italy; ^6^ Energy Control, Associació Benestar i Desenvolupament, Barcelona, Spain; ^7^ Department of Psychiatry, University of Cambridge/Cambridgeshire and Peterborough NHS Foundation Trust, Cambridge, United Kingdom

**Keywords:** JWH-122, JWH-210, synthetic cannabinoid receptor agonists (SCRAs), physiological effects, subjective effects

## Abstract

Synthetic cannabinoids (SCs) are a group of new psychoactive drugs used recreationally with potential health risks. They are monitored by the EU Early Warning System since 2010 due to severe adverse effects on consumers. JWH-122 and JWH-210 are naphthoylindole SCs and potent cannabinoid receptor CB1 and CB2 agonists. Information about the effects of SCs usually is available from intoxication cases and surveys, and few studies on humans after controlled administration or observational/naturalistic studies using standardized measures of cardiovascular and subjective effects are available. The aim of this study was to evaluate the acute pharmacological effects of JWH-122 and JWH-210 recreational consumption in a 4 h observational study and assess their disposition in oral fluid (OF). Sixteen volunteers self-administered 1 mg dose of JWH-122 (*n* = 8) or 2.25 mg mean dose of JWH-210 (range 2–3 mg, *n* = 8) by inhalation (smoking). Physiological parameters including blood pressure (systolic and diastolic), heart rate (HR), and cutaneous temperature were measured. A set of visual analog scales, the 49-item short-form version of the Addiction Research Center Inventory (ARCI), and the Evaluation of the Subjective Effects of Substances with Abuse Potential (VESSPA-SSE) were used for the evaluation of subjective effects. OF was collected at baseline and at 10, 20, and 40 min and 1, 2, 3, and 4 h after self-administration. Statistically significant increases in systolic blood pressure (SBP), diastolic blood pressure (DBP), and HR were observed after JWH-122 self-administration but not after JWH-210 self-administration. JWH-210 self-administration produced significant changes in subjective drug effects, similar to those induced by THC (intensity, high, good effects, and hunger). The subjective effects following JWH-122 consumption were minimal. The maximal effects were mostly observed 20 min after intake. JWH-122 and JWH 210 OF concentration reached a peak 20 min after administration and could not be detected after 3 h. The results demonstrated a different pattern of effects of these two SCs. Due to the limitations of our observational study, further research with a larger sample and controlled studies are needed to better define the acute pharmacological effect and health risk profile of JWH-122 and JWH-210.

## Introduction

Synthetic cannabinoids (SCs), also known as “synthetic cannabinoid receptor agonists” (SCRAs), are a chemically diverse group of small nonpolar and lipid-soluble molecules functionally similar to delta-9-tetrahydrocannabinol (THC) with a higher binding affinity for cannabinoid receptors CB1 and CB2 than that of natural cannabinoids, such as THC (Huffman et al., 2003; Huffman et al., 2005). Initially, many SCs were developed for research purposes to investigate the endocannabinoid system or as potential therapeutic drugs. More recently, SCs have been diverted for recreational purposes, and new molecules emerged (Pertwee, 2006). SCs usually are named after the scientist/institution/company who first synthesized (JWH, CP, HU, AM, WIN, and RCS series) the substance or take names helping their marketing. From the early 2000s, SCs were introduced onto the drug market as “legal highs,” “herbal incense,” “K2,” or “Spice” as a legal alternative to natural cannabis (ElSohly et al., 2014; Kemp et al., 2016; Ernst et al., 2011). Currently, SCs are the largest and most structurally diverse class of new psychoactive substances (NPS) seized in Europe, with at least 190 substances monitored by the EU Early Warning System since 2010 (European Monitoring Centre for Drugs and Drug Addiction and Europol, 2019; European Monitoring Centre for Drugs and Drug Addiction, 2020).

Due to their high affinity for the CB1 receptor (Huffman et al., 2005), SCs usually are more potent than natural cannabinoids, with potential health risks. The pattern of acute clinical toxicity associated with the recreational use of SCs is frequently characterized by tachycardia, agitation, and nausea, which typically resolve with symptomatic care (Cohen et al., 2019; Tournebize et al., 2017; Hermanns-Clausen et al., 2013). In some cases, severe toxicity including stroke, seizure, myocardial infarction, rhabdomyolysis, acute kidney injury, psychosis, suicidal ideation, and hyperemesis has been associated with SCs use, and SCs have been directly or indirectly involved in fatal cases (Hermanns-Clausen et al., 2013; Tait et al., 2016).

JWH-122 and JWH-210 are naphthoylindolic compounds and act as potent cannabinoid agonists at CB1 and CB2 receptors. According to a structure-activity relationship study, JWH-122 and JWH-210 have a binding affinity at cannabinoid CB1 receptors 60 and 90 times higher than that of THC, respectively. JWH-122 and JWH-210 CB2 receptors’ affinity is 30 and 50 times higher than that of THC, respectively (Huffman et al., 2003; Huffman et al., 2005).

Recently, a rise in the recreational use of extremely potent SCs such as JWH-122 and JWH-210 has been reported (La Maida et al., 2020). To date, the recreational use of JWH-122 and/or JWH-210 has been analytically documented in several cases of driving under the influence (DUI) and nonfatal and fatal cases (Yeakel and Logan, 2013; Dziadosz et al., 2014; Musshoff et al., 2014; Tuv et al., 2014). Hallucinations, disorientation, agitation, tachycardia, and/or hypertension in intoxication cases involving JWH-122 have been described (Coppola et and Mondola, 2017).

Most data of the pharmacological effects are based on surveys and series or cases of acute intoxications (Hermanns-Clausen et al., 2013; Hermanns-Clausen et al., 2016). There is a lack of studies in humans evaluating the acute pharmacological effects of SCs in experimental or observational controlled settings (Theunissen et al., 2018; La Maida et al., 2021). New controlled research is needed to understand the complex pharmacological effects of this group of substances.

The aim of this study was to evaluate the acute pharmacological effects and time course (TC) concentrations of JWH-122 and JWH-210 in oral fluid (OF) in an observational study.

## Materials and Methods

### Participants

Sixteen healthy subjects participated in the study. Eight subjects (one female and seven males) self-administrated JWH-122 and eight subjects (three females and five males) self-administrated JWH-210. Participants were recreative drug users and had used cannabis and SCs at least once in their lifetimes without experiencing serious adverse reactions. Exclusion criteria were history of any serious medical or mental disorder including drug dependence (except for nicotine), use of chronic medication, and serious adverse reactions with cannabis and/or SCs. Subjects were recruited through word-of-mouth and snowball sampling *via* the nongovernmental Organization Energy Control (ABD). The study protocol was submitted and approved by the Clinical Research Ethics Committee of our center, Germans Trias i Pujol University Hospital (CEI HUGTiP, Barcelona, Spain; ref. PI-18-267), and was conducted according to the Declaration of Helsinki recommendations. All the participants were correctly and fully informed, both orally and in writing, of the purpose, methods, and means of the study. All the subjects agreed to participate in the study and signed informed consent prior to inclusion. Participants received monetary compensation for their participation.

### Study Design and Treatments

The study design was naturalistic, prospective, and observational, with minimal intervention. The self-selected dose of JWH-122 or JWH-210 was mixed with 1 g tobacco and prepared as a joint. The participants had a maximal time of 5 min to smoke the joint following the subject usual habits of consumption (3–4.5 min, 10–11 inhalations). The mouth of the participants was washed with plain water to reduce contamination that could interfere with OF sampling (de Castro et al., 2014). All the doses that were self-administrated were also self-selected by each participant based presumably on their previous experience. Subjects brought their powder of SCs to the testing site themselves, which they had obtained from an unknown source. Although no information was available about the synthesis of the drug, those samples were tested by Energy Control, a harm reduction organization that provides a Drug Checking Service for users. The JWH-122 and JWH-201 contents were analyzed with gas chromatography associated with mass spectrometry (GC/MS) and showed that the substance purity was more than 95% with no toxic adulterants. The absence of the most common drugs of abuse including cocaine, MDMA, amphetamine, methamphetamine, 2C-B, other phenethylamines, heroin, LSD, DMT, other tryptamines, ketamine, psilocybin, salvinorin A, natural, SCs, and most of the NPS was analytically confirmed (Grifell et al., 2017; Graziano et al., 2019). The dose of JWH-122 and JWH-210 was selected after reviewing the literature and drug users fora (Hermanns-Clausen et al., 2013; Tournebize et al., 2017). Similar doses were reported in JWH-122 and JWH-210 users by Energy Control, which recommends subjects to take from 1 mg JWH-122 and 2–3 mg JWH-210 to avoid possible health risks.

The mean JWH-122 dose was 1 mg (all subjects self-administrated 1 mg, *n* = 8) and the mean JWH-210 dose was 2.25 mg (one male and one female self-administrated a 3 mg dose, and four males and two females self-administrated 2 mg).

### Procedures

All participants underwent a general medical examination and a psychiatric evaluation prior to the study sessions. They received training to fill the questionnaires and follow the procedures employed in the study. Sessions took place at a private club with ambient music, and participants could talk, read, or play table games and interact during the sessions, but not during the evaluation time. Sessions were planned on two different days, each day for one of the two groups of volunteers per substance. On the day of the session, subjects were admitted to the selected recreational venue and they were questioned about any event that could impede their participation. They were asked to refrain from taking drugs two days prior to the session, and alcohol concentrations in expired air were measured before the beginning of the sessions. Assessments were performed at baseline (before dose) and at 10, 20, and 40 min and 1, 2, 3, and 4 h after self-administration. Urine spot samples were collected prior to the administration, to exclude substance drug use (benzodiazepines, barbiturates, morphine, cocaine, amphetamines, methamphetamines, MDMA, marijuana, and phencyclidine) with One Step Rapid Test 10 Test Drug Screen (GIMA, Gessate, Milan, Italy). At baseline, no check for recent SC’s use was performed because there are no rapid tests for SC detection available. However, an OF sample was collected at baseline (before administration) in which no traces of JWH-210, JWH-122, or other SCs were detected. Tobacco users were asked to refrain from smoking 2 h before the beginning of the study session until the end of the session. Adverse events were assessed during the study sessions and were reported within 24 h after the self-administration session (24 h by a phone call).

### Physiological Effects

Noninvasive systolic blood pressure (SBP), diastolic blood pressure (DBP), and heart rate (HR) were determined with an Omron® monitor at baseline and at 10, 20, and 40 min and 1, 2, 3, and 4 h after administration. The oral temperature was measured at the same time.

### Subjective Effects

Subjective effects were measured at different moments during the session, using a set of visual analog scales (VASs), the 49-item Addiction Research Center Inventory (ARCI) form, and the Evaluation of Subjective Effects of Substances with Abuse Potential questionnaire (VESSPA-SP).

VAS from “not at all” to “extremely” were used to rate some items such as “intensity,” “high,” “good effects,” “bad effects,” “hunger,” “drowsiness,” “dizziness,” “confusion,” “nausea,” “vomits,” “anxiety,” “aggressiveness,” “hallucinations-seeing of lights or spots,” “hallucinations-hearing sounds or voices,” and “hallucinations-seeing animals, things, insects or people” (Papaseit et al., 2018; Papaseit et al., 2020).

The Spanish validated version of the short-form ARCI is a true/false 49-item questionnaire, an instrument for the determination of subjective drug effects (Lamas et al., 1994). It includes five subscales related to drug sedation (pentobarbital-chlorpromazine-alcohol group, PCAG), euphoria (morphine-benzedrine group, MBG), dysphoria and somatic symptoms (lysergic acid diethylamide group, LSD), intellectual efficiency and energy (benzedrine group, BG), and d-amphetamine-like effects (amphetamine, A).

The VESSPA is a questionnaire measuring the changes in subjective effects caused by different drugs, including stimulants and psychedelics. It includes six subscales: sedation (S), psychosomatic anxiety (ANX), changes in perception (CP), pleasure and sociability (SOC), activity and energy (ACT), and psychotic symptoms (PS) (González et al., 2015).

The VASs were administered at baseline and at 10, 20, and 40 min and 1, 2, 3, and 4 h after drug administration. ARCI and VESSPA forms were completed at baseline and at 1, 2, 3, and 4 h after drug administration.

### OF Concentrations

OF was collected with Salivette® tubes at baseline and at 10, 20, and 40 min and 1, 2, 3, and 4 h after self-administration.

Samples were then centrifuged and frozen at −20°C until analysis. JWH-210 and JWH-122 were quantified by a modified and validated liquid chromatography-mass spectrometry method (LC-MS/MS) (Pellegrini et al., 2020).

### Statistical Analysis

Physiological (SBP, DBP, HR, and cutaneous T) and subjective (VAS, ARCI, and VESSPA) variables were compared to baseline. Peak effects (Emax) were determined and the area under the curve of the effects (AUC0−4 h) was calculated using the trapezoidal rule by the Pharmacokinetic Functions for Microsoft Excel (Joel Usansky, Atul Desai, and Diane Tang-Liu, Department of Pharmacokinetics and Drug Metabolism, Allergan, Irvine, CA, United States).

For statistical analysis, four consecutive strategies were performed. Firstly, since subjects selected two different JWH-210 doses (2 and 3 mg, see *Study Design and Treatments*), a one-way analysis of variance (ANOVA) test was conducted to evidence the possible influence of JWH-210 dose on the different variables evaluated. The results showed no statistically significant differences between the two doses. Hence, the presented statistical analysis was performed considering data as just one dose. Secondly, Student’s *t*-test for unpaired samples was performed to compare Emax and AUC0−4 h of JHW-122 and JWH-210 of all parameters calculated. Differences in time to reach peak effects (Tmax) between JHW-122 and JWH-210 were assessed using a nonparametric test (Wilcoxon test). Thirdly, a GLM two-way repeated-measures ANOVA with drug (JWH-122 and JWH-201) and time (baseline, 10, 20, and 40 min, and 1, 2, 3, and 4 h) as factors was used to compare the TC of effects between JHW-122 and JWH-210. If significant differences were detected in drug and time factor, a sequential Bonferroni correction was applied to correct for multiple comparisons. Fourthly, since no control group was included, Dunnett’s multiple comparison post hoc test was conducted to evaluate time effects for each SC (JWH-122 and JWH-2010) comparing the different time points with baseline values (comparing times 0–10, 0–20, and 0–40 min and 0–1, 0–2, 0–3, and 0–4 h). Statistical analyses were performed using PAWS Statistics version 18 (SPSS Inc., Chicago, IL, United States). Statistical significance was defined as *p* < 0.05.

For OF data, only a descriptive analysis was presented showing main pharmacokinetics data, e.g., maximum concentration (Cmax), the time needed to reach the maximum concentration (Tmax), and AUC0–4. These descriptive parameters were calculated using the Pharmacokinetic Functions for Microsoft Excel (Joel Usansky, Atul Desai, and Diane Tang-Liu, Department of Pharmacokinetics and Drug Metabolism, Allergan, Irvine, CA, United States). No statistical analysis was performed between OF concentrations measured comparing both SCs.

## Results

### Participants

Sixteen polydrug recreational users who reported previous multiple experiences with cannabis and having used SCs at least once in their lives were recruited for the study (4 females and 12 males). Eight subjects were self-administrated by inhalation 1 mg of JWH-122 (seven males and one female). They had a mean age of 30 ± 6 years (range: 23–41 years), weighing 72.95 ± 11.22 kg (range: 60–96 kg), and their mean body mass index (BMI) was 24.16 ± 4.09 kg/m^2^ (range: 19.59 ± 32.45 kg/m^2^). Six participants were current tobacco smokers.

Eight subjects self-administrated JWH-210 by inhalation. One male and one female self-administrated a 3 mg dose, and four males and two females self-administrated 2 mg. They had a mean age of 31 ± 7 years (range: 22–41 years), weighing 69.63 ± 13.38 kg (range: 54–93 kg), and their mean BMI was 23.26 ± 2.49 kg/m^2^ (range: 18.29–26.04 kg/m^2^). Five participants were current tobacco smokers (Table 1).

**TABLE 1:** Summary of sociodemographic data and recreative drug use for the participants.

	JWH-122 (*n* = 8)	JWH-210 (*n* = 8)
	Mean ± SD (range)	Mean ± SD (range)
**Sociodemographic data**		
Female/male	1/7	3/5
Age (years)	30 ± 6 (23–41)	31 ± 7 (22–41)
Weight (kg)	72.95 ± 11.22 (60–96)	69.63 ± 13.38 (54–93)
Height (m)	1.74 ± 0.05 (1.65–1.80)	1.73 ± 0.12 (1.50–1.89)
BMI (kg/m^2^)	24.16 ± 4.09 (19.59–32.45)	23.26 ± 2.49 (18.29–26.04)
**Recreative drug use**		
Alcohol (g/day)	9.68 ± 10.15 (1.43–32)	15.86 ± 16.72 (2.86–48)
Tobacco (cig/day)	8.10 ± 5.88 (2–16) (*n* = 6)	8.10 ± 5.34 (0.5–15) (*n* = 5)
Cannabis (joints/day)	0.46 ± 0.27 (0.10–0.73)	0.10 ± 0.06 (0.03–0.17)
MDMA^a^	0.63 ± 1.41 (0–4)	0.0 ± 0.0 (0–0)
Amphetamine^a^	0.38 ± 0.74 (0–2)	0.13 ± 0.35 (0–1)
NPS/legal highs^a^	0.75 ± 1.75 (0–5)	0.13 ± 0.35 (0–1)
Cocaine^a^	0.5 ± 1.07 (0–3)	0.0 ± 0.0 (0–0)
Hallucinogens^a^	0.63 ± 1.41 (0–4)	0.63 ± 1.0 (0–3)

a Number of times used during the previous month.

### Physiological Effects

**FIGURE 1:**
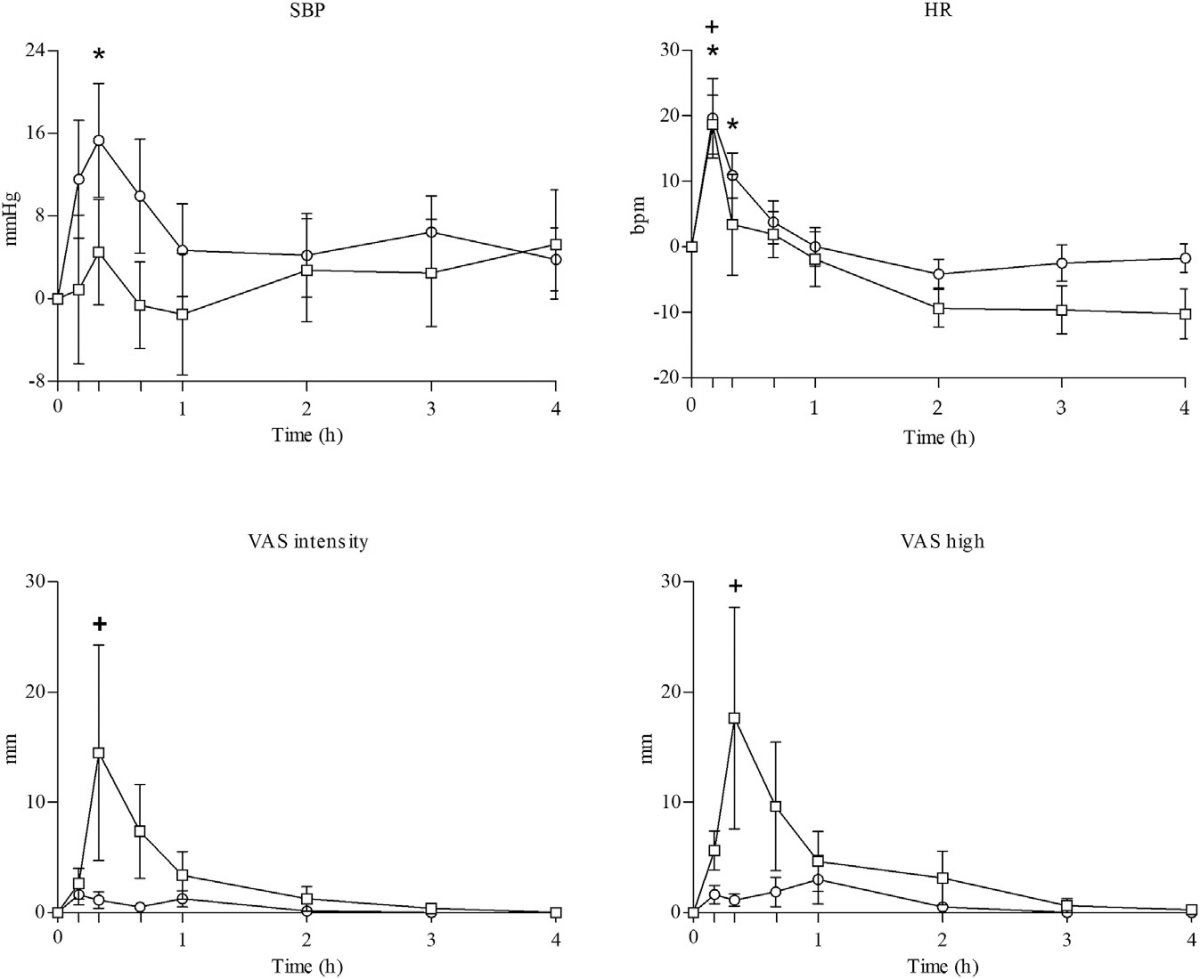
Summary of the course of main physiological (systolic blood pressure and heart rate) and subjective effects (intensity and high in visual analog scales) observed after administration of JWH-122 (*n* = 8) and JWH-210 (*n* = 8). ○ JWH-122; □ JWH-210; values are mean and standard error. Symbols indicate a significant difference to baseline: for JWH-122 * (*p* < 0.05); for JWH-210 + (*p* < 0.05), **+** (*p* < 0.05).

JWH-122 and JWH-210 effects on physiological variables are presented in Table 2 and Figure 1.

**TABLE 2:** Summary of significant statistical results on the physiological and subjective effects observed after administration of JWH-122 (*n* = 8) and JWH-210 (*n* = 8).

	Parameter	Mean ± SD		Student’s *t*-test comparison effects of JWH-122 vs. JWH-210		ANOVA comparison time course effects of JWH-122 vs. JWH-210			Dunnett’s test comparison to baseline	
		JWH-122	JWH-210	T (df = 14)	*p*- Value	F (df = 7,98)	*p*- Value	TC points	JWH-122	JWH-210
**Physiological effects**										
SBP (mmHg)	E_max_	23.68 ± 14.50	4.87 ± 23.91	1.902	0.078					
	AUC_0-4h_	24.66 ± 30.63	7.90 ± 53.90	0.764	0.457					
	TC					1.094	0.373	NS	b	NS
DBP (mmHg)	E_max_	8.81 ± 15.62	11.43 ± 12.85	-0.367	0.719					
	AUC_0-4h_	22.16 ± 23.52	18.10 ± 35.66	0.269	0.792					
	TC					1.951	0.070	NS	a, c	NS
HR (bpm)	E_max_	16.37 ± 21.69	-0.37 ± 28.60	1.320	0.208					
	AUC_0-4h_	−0.34 ± 24.34	−20.85 ± 390.6	1.261	0.228					
	TC					0.713	0.661	NS	a, b	a
T (ºC)	E_max_	0.15 ± 0.12	0.05 ± 0.33	0.807	0.433					
	AUC_0-4h_	0.23 ± 0.33	−0.06 ± 0.39	1.649	0.121					
	TC					3.054	0.006	**1**, 4	NS	NS
**Subjective effects**										
Intensity (mm)	E_max_	2.75 ± 2.66	16.5 ± 27.02)	−1.432	0.174					
	AUC_0-4h_	1.67 ± 1.98	10.34 ± 18.65	−1.308	0.212					
	TC					1.801	0.095	NS	NS	b
High (mm)	E_max_	5.87 ± 5.98	19.00 ± 27.79	−1.306	0.213					
	AUC_0-4h_	3.68 ± 4.70	15.44 ± 26.77	−1.224	0.241					
	TC					2.283	0.034	NS	NS	b
Hunger (mm)	E_max_	6.50 ± 11.13	29.70 ± 26.91	−2.258	0.040					
	AUC_0-4h_	10.41 ± 22.42	35.10 ± 30.35	−1.850	0.085					
	TC					4.189	<0.001	4	NS	**g**
**Addiction Research Center Inventory (ARCI) form**										
MBG (euphoria) (score)	E_max_	1 ± 1.41	1.37 ± 2.38	−0.382	0.708					
	AUC_0-4h_	1.25 ± 1.83	2.37 ± 3.74	−7.64	0.457					
	TC					0.237	0.916	NS	NS	d
BG (intellectual efficiency and energy) (score)	E_max_	0.5 ± 0.53	−0.25 ± 1.58	1.271	0.224					
	AUC_0-4h_	0.37 ± 0.51	−0.31 ± 1.79	1.043	0.315					
	TC					2.986	0.026	NS	NS	NS
A (amphetamine-like effects) (score)	E_max_	1.25 ± 1.38	0.75 ± 0.88	0.858	0.405					
	AUC_0-4h_	1.25 ± 1.38	1.68 ± 2.10	−0.491	0.693					
	TC					2.350	0.065	NS	NS	d
**Evaluation of subjective effects of substances with abuse potential questionnaire (VESSPA-SP)**										
ANX (psychosomatic anxiety) (score)	E_max_	0.12 ± 0.17	0.43 ± 0.28	−2.669	0.018					
	AUC_0-4h_	0.19 ± 0.36	0.78 ± 0.68	−2.116	0.053					
	TC					4.463	0.003	1	NS	NS

Emax = peak effects 0–4 h (differences from baseline); AUC0−4 h = area under the curve from 0 to 4 h; TC = temporal course from 0 to 4 h. Emax measured by mmHg [SBP and DBP (systolic blood pressure and diastolic blood pressure)], bpm [HR (heart rate)], °C [T (cutaneous temperature)], mm [visual analog scale (VAS)], and score Addiction Research Center Inventory (ARCI) and expressed as mean and standard deviation. For Emax and AUC0−4, a Student’s *t*-test for independent sample was used (see statistical analysis). A *p*-value < 0.05 was considered statistically significant. For TC, a ANOVA was used to measured differences between JWH-120 and JWH-210 and statistical differences are presented as “time point” (*p* < 0.05) and “time point” (*p* < 0.01). For Dunnett’s test, statistical differences are presented as “a” *p* < 0.05, “a” *p* < 0.01 (times 0–10 m), “b” *p* < 0.05, “b” *p* < 0.01 (times 0–20 min), “c” *p* < 0.05, “c” *p* < 0.01 (times 0–40 min), “d” *p* < 0.05, “d” *p* < 0.01 (times 0–1 h), “e” *p* < 0.05, “e” *p* < 0.01 (times 0–2 h), “f” *p* < 0.05, “f” *p* < 0.01 (times 0–3 h), and “g” *p* < 0.05, “g” *p* < 0.01 (times 0–4 h) (see statistical analysis). df = degrees of freedom.

Self-inhalation of JWH-122 and JWH-210 produced changes in the physiological variables taken into consideration. Compared to baseline, JWH-122 self-inhalation produced statistically significant increases in SBP, DBP, and HR and JWH-210 self-administration only increased HR. The maximal effects were observed at 10–20 min following administration. No statistically significant differences were found between JWH-122 and JWH-210 in terms of Emax, AUC0–4 h, or TC points, except for the body temperature (Tº) at 1 and 4 h.

### Subjective Effects

JWH-122 and JWH-210 effects on subjective variables are presented in Table 2 and Figure 1.

Self-inhalation of JWH-122 and JWH-210 induced some changes in subjective effects.

JWH-122 self-inhalation increased VAS “intensity,” “high” and “good effects,” “hunger,” “somnolence,” “dizziness,” “confusion,” and “anxiety,” and JWH-210 increased VAS “intensity” and “high” and “good effects.” For both SCs, maximal VAS effects were mostly observed 20 min after intake. Compared to baseline, statistically significant increases were detected on VAS “intensity,” “high effects,” and “hunger” for JWH-210 only. When comparing JWH-122 and JWH-210, no statistically significant differences were found for Emax or AUC0–4 h. For TC, differences between SCs were only detected for VAS “hunger” at 4 h.

Self-inhalation of JWH-122 or JWH-210 produced slight changes in the majority of ARCI questionnaire subscales, with more marked increases in the subscales sedation (PCAG: pentobarbital chlorpromazine-alcohol group), euphoria (MBG), and amphetamine (A). Compared to baseline, statistically significant increases were found for MBG and A following self-inhalation of JWH-210.

Compared to baseline, JWH-122 and JWH-210 induced nonsignificant increases in the VESSPA-SP questionnaire. When comparing JWH-122 and JWH-210, no statistically significant differences were found for Emax or AUC0–4 h. For TC, differences between SCs were only detected for “ANX” subscale at 1 h.

### Adverse Events

All the selected doses were well tolerated with no relevant adverse events during the study session. Within 24 h after the self-administration, one subject who self-administrated JWH-122 reported faintness and three subjects who self-administrated 2 mg of JWH-210 reported 1) hypotension, 2) headache, dizziness, and vomit, and 3) self-limited mild stabbing chest pain, respectively. All resolved spontaneously, and the participants did not need medication or medical assistance.

### JWH-122 and JWH-210 OF Concentrations

**FIGURE 2:**
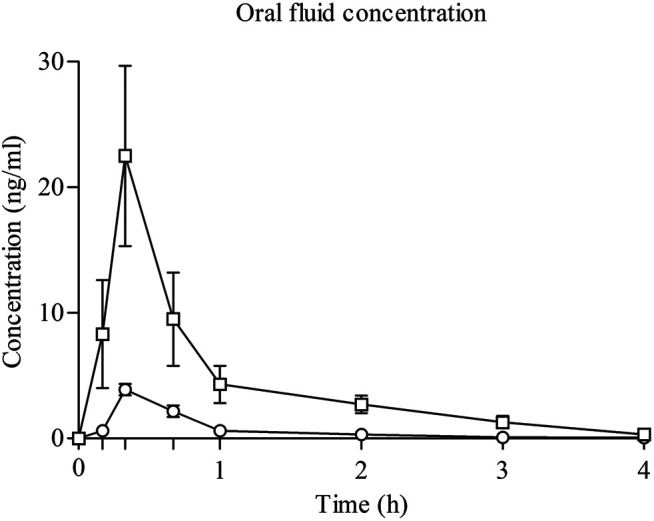
Time course of JWH-122 (*n* = 8) and JWH-210 (*n* = 7) concentrations in oral fluid. ○ JWH-122; □ JWH-210; values are mean and standard error.

JWH-122 and JWH-210 OF concentration profiles over time (no data available from one subject consuming JWH 210) are shown in Figure 2. JWH-122 OF concentration reached a peak 20 min after administration with a mean maximum concentration (Cmax) of 3.88 ng/ml (SD ± 1.23), decreased to a mean value of 0.62 ng/ml at 1 h, and was not detected after 3 h. Mean AUC0–4 h was 2.60 ng/mlh (SD ± 0.81). Similarly, JWH-210 OF concentration peaked 20 min after inhalation and was not detected after 3 h. JWH-210 Cmax was 22.54 ng/ml (SD ± 19.02) 20 min after intake and dropped to a mean value of 4.30 ng/ml at 1 h. AUC0–4 h mean value was 17.18 ng/ml h (SD ± 12.51).

## Discussion

To the best of our knowledge, controlled studies on JWH-122 and JWH-210 pharmacology and pharmacokinetics in humans are lacking. To date, this is the first study assessing the acute pharmacological effects and JWH-122 and JWH-210 OF concentrations in recreational users. In our study, 1 mg JWH-122 inhalation produced significant acute cardiovascular effects (SBP, DBP, and HR) with minimal changes in subjective effects (not statistically significant). In the same naturalistic setting, the inhalation of a recreational JWH-210 dose (2–3 mg) induced a pattern of acute effects characterized by a very mild increase of DBP and HR (not statistically significant) and significant increases in subjective effects. The global magnitude of response attributable to JWH-122 and JWH-210 is lower than that of cannabis. The results obtained can be compared with those observed in a similar recent study evaluating the effects of UR-144 consumption, as compared to those of THC after inhaled administration. JWH-122 and UR-144 cardiovascular and subjective effects were similar in magnitude (La Maida et al., 2021). UR-144 cardiovascular effects were slightly higher than those induced by JWH-210, but JWH-210 subjective effects were higher. When compared to 20 mg THC, all three SCs induced less intense cardiovascular and subjective effects (La Maida et al., 2020). JWH-210 results are in agreement with the marked but transient cardiovascular and euphoric effects described following the controlled administration by inhalation of JWH-018, one of the most studied compounds of the JWH family (Theunissen et al., 2018; Toennes et al., 2018; Theunissen et al., 2019; Theunissen et al., 2021). Regarding the subjective effects, some subjects reported moderate to high punctuations in bad effects after JWH-122 (2 subjects) and JWH-210 (1 subject) intake. According to the literature, “disliking effects” are a well-known effect reported mainly by occasional SCs users, while frequent users more likely reported liking effects (Cooper, 2016). In addition, neutral response, no response, and no adverse events are also more likely reported by single or several time SCs users. During the study, hunger was the most reported nondesirable effect for all the subjects who self-inhaled SCs. Adverse events reported 24 h after JWH-122 and JWH-210 self-administration were limited to slight-mild cardiovascular and/or gastrointestinal symptoms and headache not requiring emergency attention in any case. These results are in line with previous retrospective observational studies reporting tachycardia, hypertension, and other electrocardiographic changes (Hermanns-Clausen et al., 2013; Hermanns-Clausen et al., 2016).

Both SCs reached their maximal concentrations in OF at 20 min and 15 min after the end of the inhalation. No previous data exist on JWH-122 and JWH-210 concentrations and TC of in OF and only limited data are published on the blood concentrations in some cases of intoxications (Hermanns-Clausen et al., 2013; Cooper, 2016). In a retrospective observational case series of subjects presenting to emergency departments with an analytically confirmed intake of JWH-210 as the only SC detected in serum samples, JWH-210 concentrations ranged from 0.18 to 90 ng/ml in serum samples obtained 0.5–16 h after drug use (median 2 h) with no data about OF samples or other biological matrices (Hermanns-Clausen et al., 2016). In the case of UR-144, maximal concentrations were achieved at 20 min (La Maida et al., 2021). For JWH-018, maximal concentrations in blood and OF were reached at 5 min after inhalation and the ratio of/serum concentrations was 1.38 at the median, showing high variability within and between subjects (Theunissen et al., 2018; Toennes et al., 2018). In our study, this ratio could not be calculated since we did not collect blood.

The study has several intrinsic limitations related to its noncontrolled design (observational design and non-placebo-controlled), the relatively small number of participants that could decrease the study power in the comparisons of the two SCs, the unknown origin of the substance, the coadministration with tobacco that could have influenced some of the cardiovascular effects and adverse events reported by the participants, the limited number of time-point measures, and the lack of blood and/or other biological matrices collection. However, there are a number of strengths to remark: the participation of males and females previously experienced with inhaled cannabis and SCs, self-selection of real-life recreational dosages by the subjects according to their preferences, the real recreational setting, and the use of the validated methodology for the evaluation of acute pharmacological effects and analytic technique to determine OF concentrations.

## Conclusion

Our results showed that JWH-122 and JWH-201 exhibit THC prototypical effects on HR and blood pressure (JWH-122) and subjective effects (JWH-210) but with a lower intensity. We did not find relevant toxic effects of JWH-122 and JWH-210, probably due to the low dose administered. JWH-122 and JWH-210 were detected in OF after dosing in naturalistic conditions, confirming OF as a potential biological matrix to document SC use. Further research with a larger sample and controlled studies are needed to better define the acute pharmacological and health risk profile of JWH-122, JWH-210, and other SCs and the relevance of OF testing. This study could serve as a blueprint for follow-up research on this topic.

## Data Availability

The raw data supporting the conclusions of this article will be made available by the authors, without undue reservation.
